# Circular RNA circEVI5 functions as a miR-433 sponge to promote renal cell carcinoma progression via GBP2-mediated oncogenic signaling

**DOI:** 10.3389/fonc.2026.1754995

**Published:** 2026-07-15

**Authors:** Yang Xiong, Jin Li, Zhi-Peng Tang, Xiao Xie, Yu Liang

**Affiliations:** Department of Urology, Pingxiang People’s Hospital, Pingxiang, Jiangxi, China

**Keywords:** circEVI5, gbp2, miR-433, renal cell carcinoma, tumor progression

## Abstract

**Introduction:**

Renal cell carcinoma (RCC) is a fatal urological malignancy with limited therapeutic options and poor prognosis. Emerging evidence has shown that a number of circular RNAs (circRNAs) exert crucial effects on carcinogenesis. Nevertheless, the biological role of circEVI5 in RCC remains poorly clarified. This study aimed to elucidate the clinical significance, biological functions, and molecular mechanisms of circEVI5 in RCC pathogenesis.

**Methods:**

The expression profile of circEVI5 was first screened based on GEO and TCGA-KIRC datasets, and further verified in RCC cell lines (A498, 786-O, ACHN, CAKI-1, OSRC-2) and normal renal tubular epithelial HK-2 cells using qRT-PCR. Functional assays (CCK-8, Transwell) and a subcutaneous xenograft model were employed to assess circEVI5’s role in proliferation, migration, and invasion. RNA pull-down, RIP, and dual-luciferase reporter assays were conducted to validate interactions among circEVI5, miR-433, and GBP2. Clinical correlations were further analyzed based on TCGA datasets and immunohistochemical staining results.

**Results:**

CircEVI5 was significantly upregulated in RCC tissues (median FC=6.619; P<0.05) and cell lines, correlating with advanced tumor stage (median FC=7.691; P<0.05) and poor survival (P=0.035). Knockdown of circEVI5 via small interfering RNA inhibited cell viability, migration, and invasion *in vitro* and suppressed tumor growth *in vivo*. Mechanistically, circEVI5 acted as a cytoplasmic molecular sponge for miR-433, which was downregulated in RCC and inversely correlated with GBP2 expression. RNA pull-down and RIP assays confirmed direct binding between circEVI5 and miR-433, while luciferase assays identified GBP2 as a miR-433 target. Rescue experiments demonstrated that miR-433 inhibition or recombinant GBP2 reversed the anti-tumor effects of circEVI5 knockdown. Clinically, GBP2 overexpression in RCC tissues predicted reduced survival, and miR-433 levels were inversely correlated with both circEVI5 (P<0.0001) and GBP2 (P=0.0003).

**Discussion:**

Our findings establish circEVI5 as a novel oncogenic driver that facilitates RCC progression through the miR-433/GBP2 regulatory axis. The circEVI5/miR-433/GBP2 network represents a potential therapeutic target, and circEVI5 may serve as a prognostic biomarker for RCC patients. This study highlights the critical role of circRNA-mediated miRNA sponging in RCC pathogenesis and provides a framework for developing RNA-based therapies.

## Introduction

According to the GLOBOCAN 2022 database, a total of 434,419 new renal cell carcinoma (RCC) cases and 155,702 RCC-related deaths were reported worldwide, highlighting the substantial global disease burden of this malignancy (1). RCC accounts for approximately 3-5% of all adult cancers, constituting approximately 85% of all primary malignant renal tumors (2). The incidence of RCC is second only to prostate cancer and bladder cancer in male genitourinary tumors, although this varies according to the ethnicity and geographical location of the patients. Nephron-sparing surgery and radical nephrectomy are currently the main treatment methods for localized and locally advanced RCC. Given the development of minimally invasive radical and partial nephrectomy techniques in recent years, the 5-year survival rate following surgery for localized RCC has reached a level of 80-95% (3). However, 20-30% of these patients develop recurrence or metastasis, and once recurrence or metastasis occurs, the vast majority of patients will eventually succumb to cancer-specific death (4). Tyrosine kinase inhibitors (TKIs), mammalian target of rapamycin (mTOR) inhibitors and immune checkpoint inhibitors (ICIs) are currently recommended as systemic therapies for metastatic RCC by the National Comprehensive Cancer Network (NCCN) and European Association of Urology (EAU) guidelines. These agents can improve objective response rates and prolong progression-free survival as well as overall survival in patients (5, 6). Clinical trials of targeted therapy have been conducted globally, and several studies have publicly released their data; however, few studies have achieved favorable outcomes to date (7). Therefore, investigating the underlying molecular mechanisms of RCC and identifying novel therapeutic targets for RCC metastasis and recurrence are of crucial importance.

Circular RNAs (circRNAs) are a class of non-coding RNAs found in eukaryotic organisms that are devoid of a 5’-terminal cap and a 3’-terminal polyadenylated tail, and they form circular structures via the formation of covalent bonds. CircRNAs are resistant to RNA exonuclease, ensuring that they are more stably expressed, and that their rates of degradation are reduced. Consequently, circRNAs are more stable and conserved compared with linear RNAs. There is growing evidence to suggest that circRNAs have a significant role in the carcinogenesis and progression of numerous types of cancer, including hepatocellular carcinoma (8), gastric cancer (9), breast cancer (10), lung cancer (11) and urological malignancies (12). In a recent study, Lin and Zhi (13) identified that the expression levels of circPRELID2 in RCC tissues and cells were upregulated. Through RNA immunoprecipitation analysis and luciferase reporter and RNA pull-down assays, they confirmed that circPRELID2 could function as a sponge for the microRNA miR-22-3p to modulate the protein expression of ETS variant 1, which, in turn, further promoted the proliferation and invasion of RCC. Huang et al. (14) also found that the expression of circPDHK1 was upregulated in clear cell RCC (ccRCC) tissues, and that this was closely associated with the World Health Organization/International Society of Urological Pathology (WHO/ISUP) stage, T stage, distant metastasis, von Hippel-Lindau mutations and Ki-67 levels. Interestingly, they identified a functional internal ribosome entry site in the circPDHK1 sequence that enabled circPDHK1 to encode a functional peptide, namely PDHK1-241aa. The PDHK1-241aa peptide was then shown to promote ccRCC proliferation, migration and invasion through activating the AKT-mTOR signaling pathway. In spite of the above-mentioned studies, however, the number of published articles that have appeared on the role of circRNAs in RCC remains low.

A related study demonstrated that circEVI5 is significantly downregulated in gastric cancer, and its low expression correlates with unfavorable patient prognosis (15). However, the underlying mechanism and roles of circEVI5 in RCC have yet to be fully elucidated; therefore, the aim of the present study was to analyze the clinical significance and mechanistic role of circEVI5 in RCC. Bioinformatics screening revealed circEVI5 as a candidate oncogenic circRNA upregulated in TCGA-KIRC cohorts. Functional experiments demonstrated that circEVI5 knockdown markedly repressed the proliferative, migratory, and invasive abilities of RCC cells *in vitro* and inhibited tumor growth *in vivo*. Mechanistically, circEVI5 directly bound miR-433, a tumor-suppressive miRNA downregulated in RCC, thereby derepressing its target GBP2. Rescue assays confirmed that miR-433 inhibition or GBP2 overexpression reversed circEVI5 knockdown effects, establishing a functional circEVI5/miR-433/GBP2 axis. This study not only elucidates circEVI5 as a novel prognostic biomarker and therapeutic target but also provides insights into circRNA-mediated miRNA sponging mechanisms in RCC, bridging a critical gap between non-coding RNA biology and precision oncology.

## Materials and methods

### Patients and tissue specimens

A total of 61 pairs of RCC tissues and adjacent normal specimens were collected from patients who underwent either radical or partial nephrectomy for histologically confirmed RCC at Pingxiang People’s Hospital between 2017 and 2019. Efforts were made to ensure that the sampled tumor tissues were freed of any necrotic elements. The adjacent normal specimens were collected from the tissues located furthest from the cancer’s margin. The present study was approved by the ethics committee of Pingxiang People’s Hospital, and written informed consent was obtained from each of the patients before they were enrolled in the study. Only the patients with sufficient tissue and valid follow-up data were included. Patients who received neoadjuvant treatments such as chemotherapy, radiation therapy, targeted therapy or immunotherapy were also excluded. All the tissue specimens were immediately frozen in liquid nitrogen following surgery, and stored a -80˚C freezer prior to RNA isolation. The pathological tumor stage was examined according to the 2017 American Joint Committee on Cancer (AJCC) TNM classification system (16). The tumor size was determined by measuring the greatest dimension of the tumor. The follow-up time was defined as the interval between surgery and death, or between surgery and the last observation in the case of surviving patients.

### Cell culture and transfection

Human RCC cell lines (A498, 786-O, ACHN, OSRC-2, and CAKI-1) and the normal renal proximal tubular epithelial cell line HK-2 were obtained from the American Type Culture Collection (ATCC, Manassas, USA). All cells were cultured in RPMI-1640 medium (Gibco, Carlsbad, USA) supplemented with 10% fetal bovine serum (FBS; Gibco) and maintained at 37 °C in a humidified 5% CO_2_ incubator. To construct circEVI5 overexpression system, the full-length circEVI5 sequence was cloned into the pLCDH-ciR vector (Geneseed, Guangzhou, China). RCC cells with low circEVI5 expression were transfected with the circEVI5 overexpression plasmid or empty vector using Lipofectamine 3000 (Invitrogen, CA, USA) according to the manufacturer’s instructions. Overexpression efficiency was confirmed by qRT-PCR using divergent primers designed for the back-splice junction. For circEVI5 downregulation, small interfering RNAs (siRNAs) targeting circEVI5 (si-circEVI5) and negative control siRNA (si-NC) were synthesized by RiboBio (Guangzhou, China). miR-433 mimics, inhibitors, and their respective controls were purchased from GenePharma (Shanghai, China). Oligonucleotide sequences were as follows: si-circEVI5-1, 5’-UCAAGUACUGUACGAUCAUU-3’; si-circEVI5-2, 5’-AGUCUAGACUGAUCUACGAG-3’; si-circEVI5-3, 5’-CUACGAUACUAGUCGAUGAA-3’; si-NC, 5’-UAGCGACUAAACACAUCAA-3’; miR-433 mimics, 5’-AUCAUGAUGGGCUCCUCGGUGU-3’; and miR-433 inhibitors, 5’-ACACCAGAGGCCCAUCAUGAU-3’. Transient transfection was performed using Lipofectamine 3000 (Invitrogen, CA, USA) according to the manufacturer’s protocol. Cells were harvested 48 hours post-transfection for subsequent functional assays. Transfection efficiency was validated by quantitative real-time PCR (qRT-PCR).

### Immunohistochemical staining and evaluation

RCC tissues were also fixed in 10% formalin and embedded in paraffin, and then cut into 4µm slices. The detailed IHC experimental procedures were performed as described previously (17). All specimens were stained using the streptavidin-peroxidase (SP) method with SP ready-to-use kit (Beijing Zhongshan Golden Bridge Biotechnology, Beijing, China) according to the manufacturer’s instructions. Sections were incubated with primary antibody against GBP2 protein (1:200 dilution; Proteintech, IL, USA) at 4 °C overnight. GBP2 expression levels were evaluated independently by two pathologists using a semi-quantitative scoring system depending on the percentage of staining positive tumor cells and the staining intensity.

### Cell counting kit-8 assay

The cell viability of RCC cells were measured using cell counting kit-8 reagent (Dojindo, Kumamoto, Japan). Transfected RCC cells (3000 cells per well) were added to 96-well plates and incubated at 37 ˚C, 5% CO2, 90% humidity for 24 hours. Each group consisted of 6 wells with good cell growth and uniform cell distribution and density. After the indicated time (24, 48, 72, and 96 hours), each well of the plates were supplemented with 10 μL of the CCK‐8 solution and maintained for 2.5 hours in a 37 °C incubator. The absorbance value at 450 nm wavelength was measured by a microplate reader.

### Transwell assays

The migratory and invasive capacities of RCC cells were evaluated using Transwell chambers (Corning, NY, USA). For the migration assay, transfected cells were trypsinized 48 hours post-transfection, resuspended in serum-free RPMI-1640 medium, and seeded into the upper chamber (2 × 10^4^ cells per well). The lower chamber was filled with RPMI-1640 containing 20% FBS as a chemoattractant. For the invasion assay, the upper chamber was pre-coated with Matrigel (BD Biosciences, NY, USA) and incubated at 37 °C for 2 hours to form a matrix barrier. Cells (5 × 10^4^ cells per well) were then seeded into the Matrigel-coated upper chamber under the same conditions as the migration assay. After 24–36 hours of incubation, non-migrated/non-invaded cells on the upper membrane surface were gently removed with a cotton swab. Cells that traversed the membrane were fixed with 4% paraformaldehyde, stained with 0.1% crystal violet, and photographed under a light microscope (Olympus IX73). The number of migrated or invaded cells was quantified by counting five random fields per chamber using ImageJ software (NIH, USA).

### *In vivo* assay

To evaluate the tumorigenic role of circEVI5 *in vivo*, a xenograft tumor model was established using male BALB/c nude mice (4–5 weeks old, purchased from Vital River Laboratory Animal Technology Co., Ltd.). Mice were housed under specific pathogen-free (SPF) conditions maintained at 22 ± 1 °C with 55 ± 5% humidity and a 12-h light/dark cycle (7:00-19:00), with autoclaved bedding changed twice weekly, and provided irradiated feed and sterile water ad libitum. All animal procedures were approved by the Ethics Committee of Pingxiang People’s Hospital (Ethics ID: 2022J121-DW11) and conducted in accordance with the National Institutes of Health (NIH) Guide for the Care and Use of Laboratory Animals. A498 cells stably transfected with si-NC or si-circEVI5 were harvested, resuspended in phosphate-buffered saline (PBS), and subcutaneously injected into the flank of mice (5 × 10^6^ cells per mouse, n = 6 per group). A total of 13 mice were employed in this study, with 12 tumor-bearing mice included for final analysis and one spare mouse excluded from the study due to unsuccessful tumor formation. Tumor growth was monitored every 1 week by measuring the length (L) and width (W) with a vernier caliper, and tumor volume was calculated using the formula: V=L×W^2^/2​. After 5 weeks, mice were euthanized by cervical dislocation under anesthesia, and tumors were excised, photographed, and weighed.

### Cellular RNA fractionation assay

To determine the subcellular localization of circEVI5, cytoplasmic and nuclear RNA fractions were isolated using the PARIS™ Kit (Thermo Fisher Scientific, MA, USA) according to the manufacturer’s protocol. Briefly, RCC cells were lysed with cell fractionation buffer to separate cytoplasmic and nuclear components. RNA from each fraction was extracted using denaturing lysis buffer, followed by DNase I treatment to eliminate genomic DNA contamination. GAPDH mRNA and U6 snRNA were utilized as cytoplasmic and nuclear markers, respectively, to validate the efficiency of subcellular fractionation.

### RNA pull-down assay

To validate the direct interaction between circEVI5 and targeted miRNAs, RNA pull-down assays were performed using biotinylated DNA probes specifically targeting the back-splice junction of circEVI5. Briefly, RCC cells were transfected with 50 nM biotinylated circEVI5 probe (Sangon Biotech, Shanghai, China) or scrambled negative control probe. After 48 hours, cells were lysed in RIP buffer (Beyotime, Shanghai, China) supplemented with RNase inhibitor (Takara, Tokyo, Japan) and protease inhibitor cocktail (Roche, Basel, Switzerland). Lysates were incubated with Dynabeads™ MyOne™ Streptavidin C1 magnetic beads (Thermo Fisher Scientific, MA, USA) at 4 °C for 4 hours. The beads were washed five times with cold RIP buffer to remove nonspecific binding. RNA complexes bound to the beads were eluted using TRIzol reagent (Thermo Fisher Scientific, MA, USA), followed by RNA purification and DNase I treatment. The enrichment of miRNAs in the pulled-down RNA was quantified by qRT-PCR.

### RNA immunoprecipitation assay

To further confirm the interaction between circEVI5 and miR-433, RIP assays were performed using the Magna RIP™ RNA-Binding Protein Immunoprecipitation Kit (Merck Millipore, MA, USA) according to the manufacturer’s instructions. Briefly, RCC cells were lysed in RIP lysis buffer containing protease inhibitor cocktail and RNase inhibitor. Cell lysates were incubated with magnetic beads conjugated with anti-Ago2 antibody or negative control IgG at 4 °C for 6 hours. After washing with RIP wash buffer, RNA-protein complexes were eluted from the beads and digested with Proteinase K to remove proteins. Co-precipitated RNA was extracted using TRIzol reagent and reverse-transcribed into cDNA. The enrichment of circEVI5 and miR-433 in the immunoprecipitates was analyzed by qRT-PCR.

### Reverse transcription-quantitative PCR analysis

Total RNA was extracted from RCC tissues and cell lines that had been completely disrupted using Invitrogen^®^ TRIzol™ reagent (Thermo Fisher Scientific, MA, USA) according to the manufacturer’s instructions. A NanoDrop™ 2000 spectrophotometer (Thermo Fisher Scientific, MA, USA) was used to measure the A_260_ and A_280_ absorbance values to ensure the quality and purity of the extracted RNA. A PrimeScript RT Reagent Kit (Takara, Tokyo, Japan), containing Prime-Script RT enzyme mix I, random primer and PrimeScript buffer, was utilized to synthesize the first-strand complementary DNA (cDNA) from the enriched RNA samples. TB Green^®^ Premix Ex Taq^™^ II (Takara, Tokyo, Japan), forward primers, reverse primers and synthesized cDNA were assembled into PCR reaction mixtures for subsequent amplification using the LightCycler^®^ 480 Real-Time PCR System (Roche Diagnostics). Quantitative PCR was performed under standardized conditions: reverse transcription at 42 °C for 30 minutes followed by 85 °C for 5 minutes, initial denaturation at 95 °C for 30 seconds, 40 cycles of amplification with denaturation at 95 °C for 5 seconds and annealing/extension at 60 °C for 30 seconds, and melt curve analysis from 65 °C to 95 °C with 0.5 °C increments every 5 seconds. To ensure accurate normalization of RNA expression levels, the housekeeping gene glyceraldehyde-3-phosphate dehydrogenase (GAPDH) was used as an endogenous control. The 2^-ΔΔCq^ method was used to calculate the fold changes in relative gene expression (18). The primer sequences (**Table 1**) were synthesized by GenePharma (Shanghai, China).

**Table 1:** Sequences (5’-3’) of primers.

Primers	Forward	Reverse
circ-EVI5	AAGGAAAGCTTCAAGGACAGC	CACCTCTTGTTCTGCTCTTCG
miR-217	GCCGAGTACTGCAT	GTGCAGGGTCCGAGGT
miR-433	GCCGAGATCATGAT	GTGCAGGGTCCGAGGT
GBP2	CTGGAAGACCTGGTGGAGGT	TGGTCCAGGTCCAGGTATG
GAPDH	AGATCCCTCCAAAATCAAGTGG	GGCAGAGATGATGACCCTTTT
U6	CTCGCTTCGGCAGCACATA	AACGCTTCACGAATTTGCGT

### Luciferase reporter assay

To confirm the direct binding between circEVI5/GBP2 mRNA and miR-433, dual-luciferase reporter assays were performed. The wild-type (WT) fragment of circEVI5 or GBP2 containing the predicted miR-433 binding site was amplified by PCR and cloned into the pmirGLO vector (Promega, WI, USA) downstream of the firefly luciferase gene. A mutant (MUT) construct with deleted seed sequence was generated using site-directed mutagenesis (Agilent Technologies, CA, USA). RCC cells were co-transfected with either WT or MUT reporter plasmids (100 ng/well) and miR-433 mimics (50 nM) or negative control (miR-NC). *Renilla* luciferase plasmid (10 ng/well; Promega, WI, USA) was included as an internal control. After 48 hours, luciferase activity was measured using the Dual-Luciferase^®^ Reporter Assay System (Promega, WI, USA) on a GloMax Navigator microplate reader (Promega, WI, USA). Firefly luciferase signals were normalized to *Renilla* values for each sample.

### Western blot analysis

To assess the protein expression of GBP2, transfected RCC cells were lysed in RIPA buffer (Beyotime, Shanghai, China) containing protease inhibitor cocktail. Protein concentrations were quantified using the BCA Protein Assay Kit (Thermo Fisher Scientific, MA, USA). Equal amounts of protein (30 μg per lane) were separated by 10% SDS-PAGE and transferred onto PVDF membranes (Merck Millipore, MA, USA). After blocking with 5% non-fat milk for 1 hour at room temperature, membranes were incubated overnight at 4 °C with primary antibodies against GBP2 (1:1000; Abcam, MA, USA) and GAPDH (1:5000; Proteintech, IL, USA) as a loading control. Membranes were then washed with TBST and incubated with HRP-conjugated secondary antibodies (1:5000; Cell Signaling Technology, MA, USA) for 1 hour at room temperature. Protein bands were visualized using the ECL Prime Western Blotting Detection Reagent (GE Healthcare, Buckinghamshire, UK) and imaged on a ChemiDoc MP Imaging System (Bio-Rad, CA, USA). Band intensities were quantified using Image Lab software (Bio-Rad), and GBP2 expression levels were normalized to GAPDH.

### Bioinformatic analysis

The GSE100186 dataset was downloaded from the GEO database (https://www.ncbi.nlm.nih.gov/geo/). GEO2R was used to compare two groups of samples in order to identify differentially expressed circRNAs using the limma R package with thresholds of |log2 fold change| ≥ 1.5 and adjusted p < 0.05. The version information of GEO2R R script were R 4.2.2, Biobase 2.58.0, GEO query 2.66.0 and limma 3.54.0. The fundamental information of each circRNA was queried using the circBase database (http://www.circbase.org/). To identify potential miRNA targets of circEVI5, we employed two computational prediction databases: the Circular RNA Interactome (CircInteractome, https://circinteractome.irp.nia.nih.gov/) and the circBank Database (http://www.circbank.cn/). miRNA response elements predicted by both platforms were cross-referenced, and miRNAs exhibiting consensus predictions from both databases were selected as high-confidence candidates. Subsequently, these candidate miRNAs were further refined by cross-validation with differentially expressed miRNAs derived from The Cancer Genome Atlas (TCGA) dataset. The TargetScan (http://www.targetscan.org/), miRTarBase (https://ngdc.cncb.ac.cn/databasecommons/database/id/167), and miRDB (https://mirdb.org/) databases were used to integrated miRNA-mRNA interaction analysis. High-confidence candidate mRNAs requiring tri-database consensus prediction were further validated by TCGA dataset.

### Statistical analysis

The association between the expression levels of the circRNAs and the clinicopathological characteristics were analyzed using the Chi-square test. Survival curves were calculated using the Kaplan-Meier method, and differences in the survival curves were analyzed using the log-rank test. Multivariate Cox-regression analyses were used to evaluate the relationship between circEVI5 expression and overall survival. Student’s t-test and one-way ANOVA were used compare two or more groups for statistical analysis, respectively. Statistical analyses were performed using GraphPad Prism 8 software (Dotmatics, UK). P-values <0.05 were considered to indicate a statistically significant difference, and experimental data were obtained from three independent experiments. All data were presented as the mean ± standard deviation.

## Results

### CircEVI5 expression was elevated in the RCC tumor specimens and associated with poor prognostic significance

**Figure 1:**
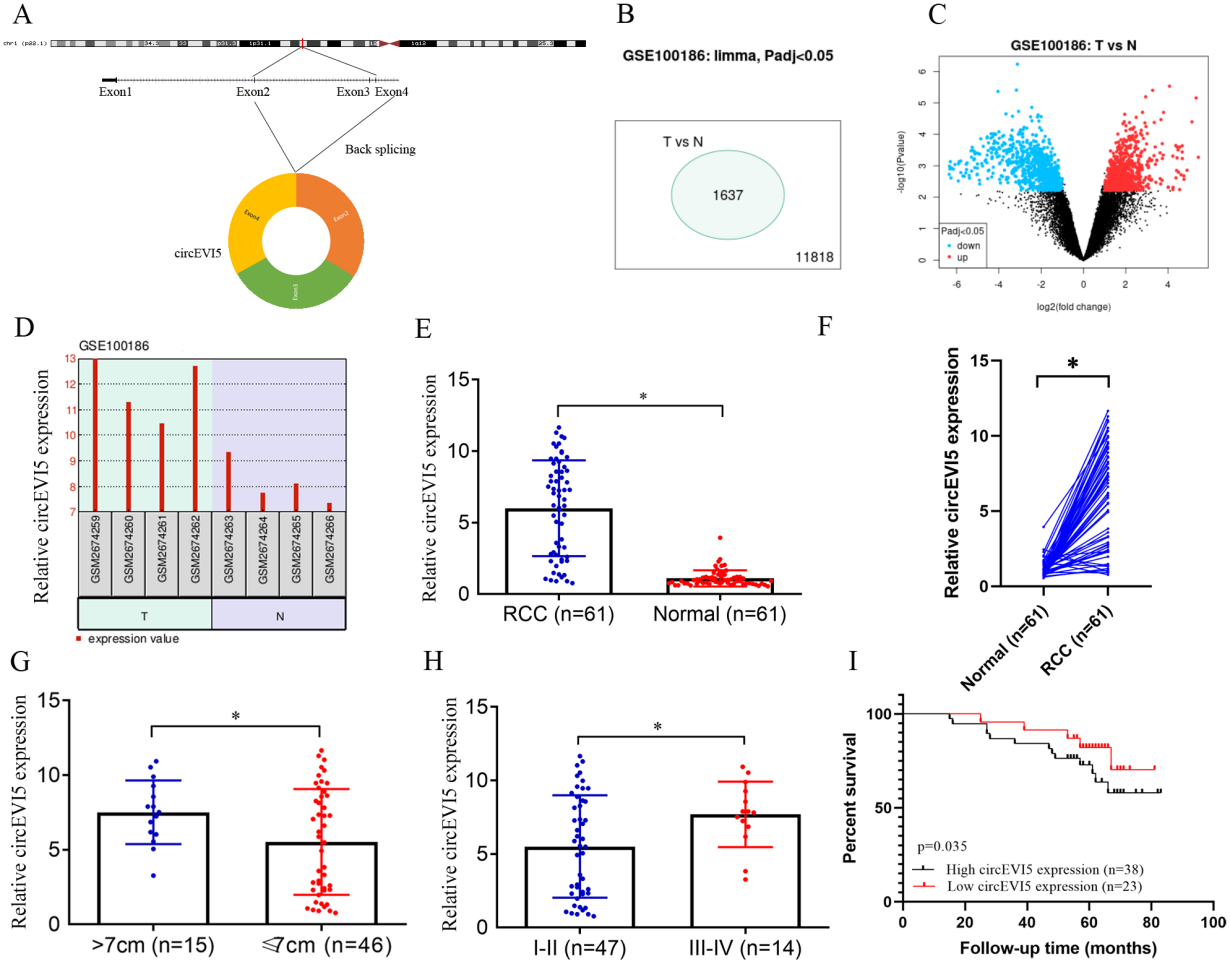
circEVI5 was overexpressed in renal cell carcinoma (RCC) tissues and associated with poor prognosis. **(A)** the schematic illustration showing the location and length of circEVI5 gene on chromosome 1 and the cyclization mechanism of circEVI5. **(B)** venn diagram generated by the limma algorithm showed that 1637 differentially expressed circRNAs were found in 11818 detected circRNAs from GSE100186 dataset. **(C)** volcano plot displayed differentially expressed circRNAs in GSE100186 RCC samples. Red dots represented the upregulated circRNAs and blue dots represented the downregulated circRNAs. **(D)** the relative circEVI5 expression values of RCC tissues and matched non-tumor tissues from GSE100186 dataset. **(E, F)** the circEVI5 expression of RCC tissues and matched non-tumor tissues from our cohort was determined by qRT-PCR. **(G, H)** the circEVI5 expression was significantly upregulated in large-volume (> 7cm) and advanced TNM stage (III-IV) RCC tissues compared to low-volume (≤ 7cm) and localized TNM stage (I-II) RCC tissues, respectively. (I) Kaplan-Meier survival analysis with log-rank testing was performed to evaluate the association between circEVI5 expression levels and clinical outcomes in RCC patients. T, RCC tissues; N, matched non-tumor tissues. *P<0.05.

CircEVI5 (circRNA ID: hsa_circ_0013162) is located at chr1:93029198‐93073284, with a full length of 339 nucleotides. It is generated via back-splicing of exons 2–4 of the EVI5 gene to form a circular structure (**Figure 1A**). A total of four RCC tissues and paired adjacent normal tissues were collected to determine the circRNA expression profiles using circRNA microarray analysis (GEO accession number: GSE100186). CircRNAs were considered to be differentially expressed when their default adjusted P-value was <0.05 and the absolute value of log_2_ FC was >1. Venn diagrams and volcano plots were subsequently used to display approximately 1,637 differentially expressed circRNAs in RCC tissues compared with adjacent normal tissues (**Figures 1B, C**). The circRNA microarray chip results revealed that the circEVI5 expression levels were markedly increased in RCC tissues compared with adjacent normal tissues (**Figure 1D**). The expression level of circEVI5 was further validated in 61 pairs of RCC tissues and adjacent non-tumor tissues using RT-qPCR analysis. In our cohort, the majority of patients (54/61, 88.5%) were diagnosed with clear cell RCC (ccRCC), whereas the remaining cases included four papillary RCC, two chromophobe RCC, and one collecting duct carcinoma, consistent with the low prevalence of these rare RCC subtypes. The results obtained showed that the expression of circEVI5 was significantly upregulated in RCC tissues (median FC = 6.619) compared with adjacent non-tumor tissues (median FC = 0.929) (**Figures 1E, F**; P < 0.05). While **Figure 1E** present this dysregulation as a box plot to illustrate population-level differences, **Figure 1F** provided paired visualization of individual samples, demonstrating consistent overexpression in tumor-normal pairs. Compared with the low-volume (≤7 cm; median FC = 5.686) and localized TNM stage RCC (I-II; median FC = 5.511) groups, a significant increase in circEVI5 expression was also observed in the high-volume (>7 cm; median FC = 7.516) and advanced TNM stage (III-IV; median FC = 7.691) RCC groups (**Figures 1G, H**; both P < 0.05). The follow-up period and median follow-up time for patients in the present study were 15-83 months and 60 months, respectively. The patients with high circEVI5 expression exhibited a significantly worse overall survival rate compared with those with low circEVI5 expression according to the Kaplan-Meier analysis (HR = 2.812, 95% CI: 1.708–10.634, *P* = 0.035; **Figure 1I**).

### Correlation between circEVI5 expression and clinicopathological parameters in 61 patients with RCC

The expression levels of circEVI5 were detected using RT-qPCR analysis in clinical samples from a total of 61 patients with RCC (29 males and 32 females). The median circEVI5 expression level was used as a cut-off to separate the samples in the high circEVI5 expression group from those in the low circEVI5 expression group. The correlations between circEVI5 levels and clinicopathological characteristics are summarized in **Table 2**. High expression of circEVI5 was identified in 62.3% (38/61) of the patients with RCC. High circEVI5 expression was also found to be significantly associated with primary tumor TNM stage (P = 0.039; OR = 0.206; 95% confidence intervals (95% CI) = 0.043-0.892) and tumor size (P = 0.004; OR = 0.078; 95% CI = 0.007-0.515), whereas no significant correlation was identified between circEVI5 expression and the patients’ age (P = 0.663; OR = 1.26; 95% CI = 0.463-3.421) or sex (P = 0.621; OR = 1.3; 95% CI = 0.49-3.638). To determine whether circEVI5 expression independently predicts prognosis in RCC, multivariate Cox regression analysis was performed, adjusting for potential confounders including age, gender, tumor size, and TNM stage. As shown in **Table 3**, high circEVI5 expression remained significantly associated with poor overall survival (HR = 4.535, 95% CI: 1.964–12.453, *P* = 0.006). Among the covariates, TNM stage (III–IV vs. I–II) was also an independent prognostic factor (HR = 0.303, 95% CI: 0.101–0.910, *P* = 0.033), whereas age, gender, and tumor size did not reach statistical significance (all *P*>0.05).

**Table 2:** Correlations between clinicopathological characteristics and circEVI5 expression in 61 renal cell carcinoma patients ^†^.

Characteristics	Total (n=61)	circEVI5 expression		χ2	P value	Odds ratio (95%CI)
		High (n=38)	Low (n=23)			
Age(years)				0.19	0.663	1.26 (0.463-3.421)
<60	34	22	12			
≥60	27	16	11			
Gender				0.244	0.621	1.3 (0.49-3.638)
Male	29	19	10			
Female	32	19	13			
Tumor size				8.158	0.004	0.078 (0.007-0.515)
≤7cm	46	24	22			
>7cm	15	14	1			
TNM stage ‡				4.243	0.039	0.206 (0.043- 0.892)
I-II	47	26	21			
III-IV	14	12	2			

CI, confidence interval.

^†^ The cases comprised: 54 clear cell carcinomas (88.5%), 4 papillary carcinomas (6.6%), 2 chromophobe carcinomas (3.3%), and 1 collecting duct carcinoma (1.6%).

^‡^ Staging was confirmed based on the 2017 AJCC TNM classification system.

**Table 3:** Multivariate Cox-regression analysis of factors associated with RCC overall survival.

Variable	Comparison	HR	95% CI	P value
Age(years)	<60 vs. ≥60	0.767	0.292-2.015	0.590
Gender	male vs. female	1.100	0.352-3.438	0.869
Tumor size	>7cm vs. ≤7cm	2.727	0.391-19.009	0.311
TNM stage	I-II vs. III-IV	0.303	0.101-0.910	0.033
CircEVI5 expression	high vs. low	4.535	1.964-12.453	0.006

RCC, renal cell carcinoma; HR, hazard ratio; CI, confidence interval.

### CircEVI5 drove malignant progression in RCC cells through potentiation of proliferative, migratory, and invasive capacities *in vitro*

**Figure 2:**
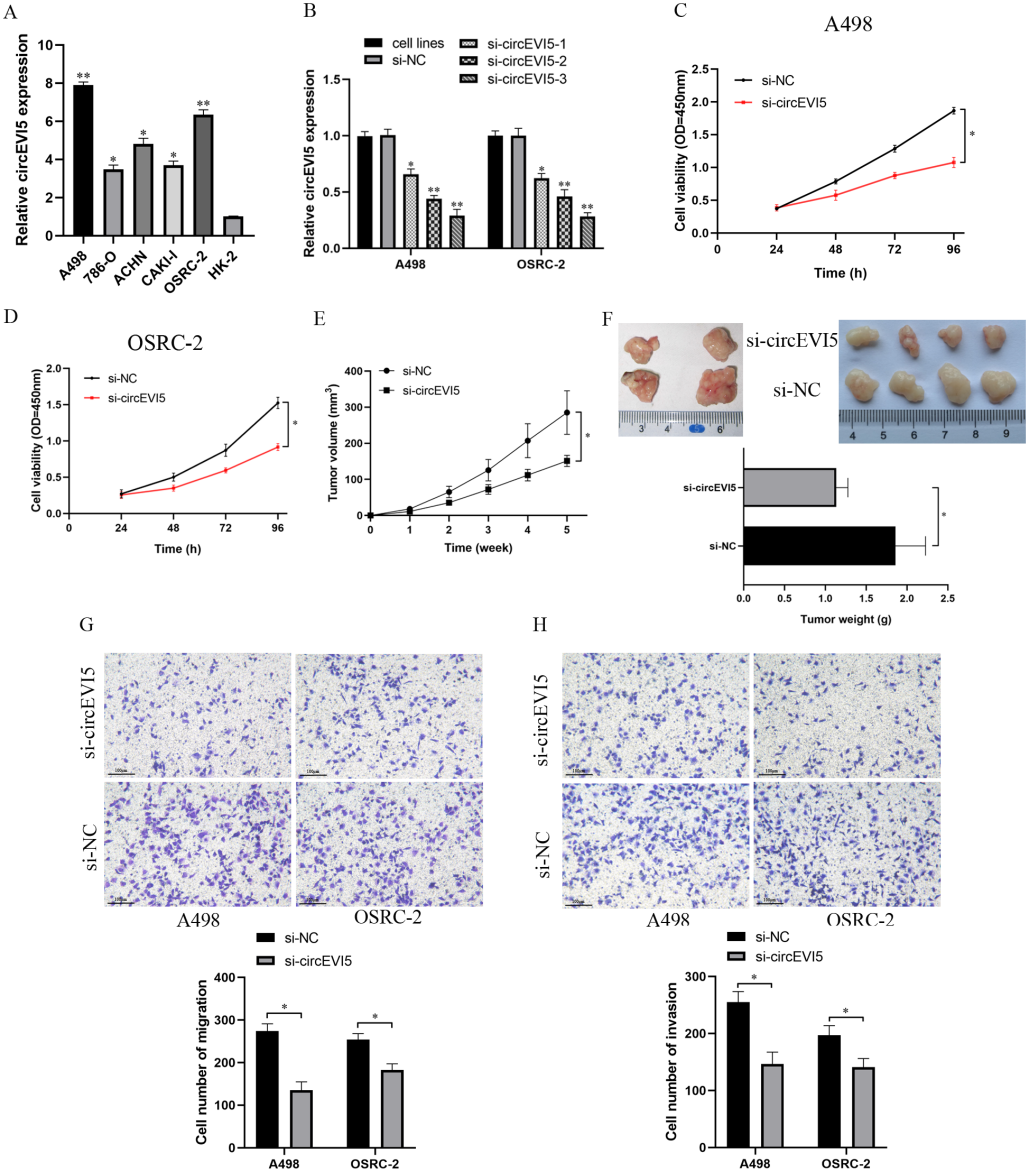
circEVI5 inhibition suppresses RCC cell proliferation and invasion *in vitro*, and attenuates xenograft tumor growth *in vivo*. **(A)** qRT-PCR was performed to measure the relative expression of circEVI5 in RCC and normal cell lines. **(B)** the relative circEVI5 expression were determined by qRT-PCR in RCC cells transfected with si-circEVI5. (**C D).** CCK‐8 assays were used to detect cell viability of transfected RCC cells. **(E)** xenograft tumor volume was measured using a vernier caliper on the indicated days. **(F)** xenograft tumors were photographed and weighed after 5 weeks of injection. Right eight tumors: initial cohort (n = 4 per group). Left four tumors: additional cohort (n = 2 per group) added upon revision to achieve a final sample size of n = 6 per group. **G,** the migration of transfected RCC cells were determined by Transwell assays. **H,** the invasion of transfected RCC cells were determined by Transwell assays. Scale bar: 100 μm. *P<0.05, **P<0.01.

**Figure 3:**
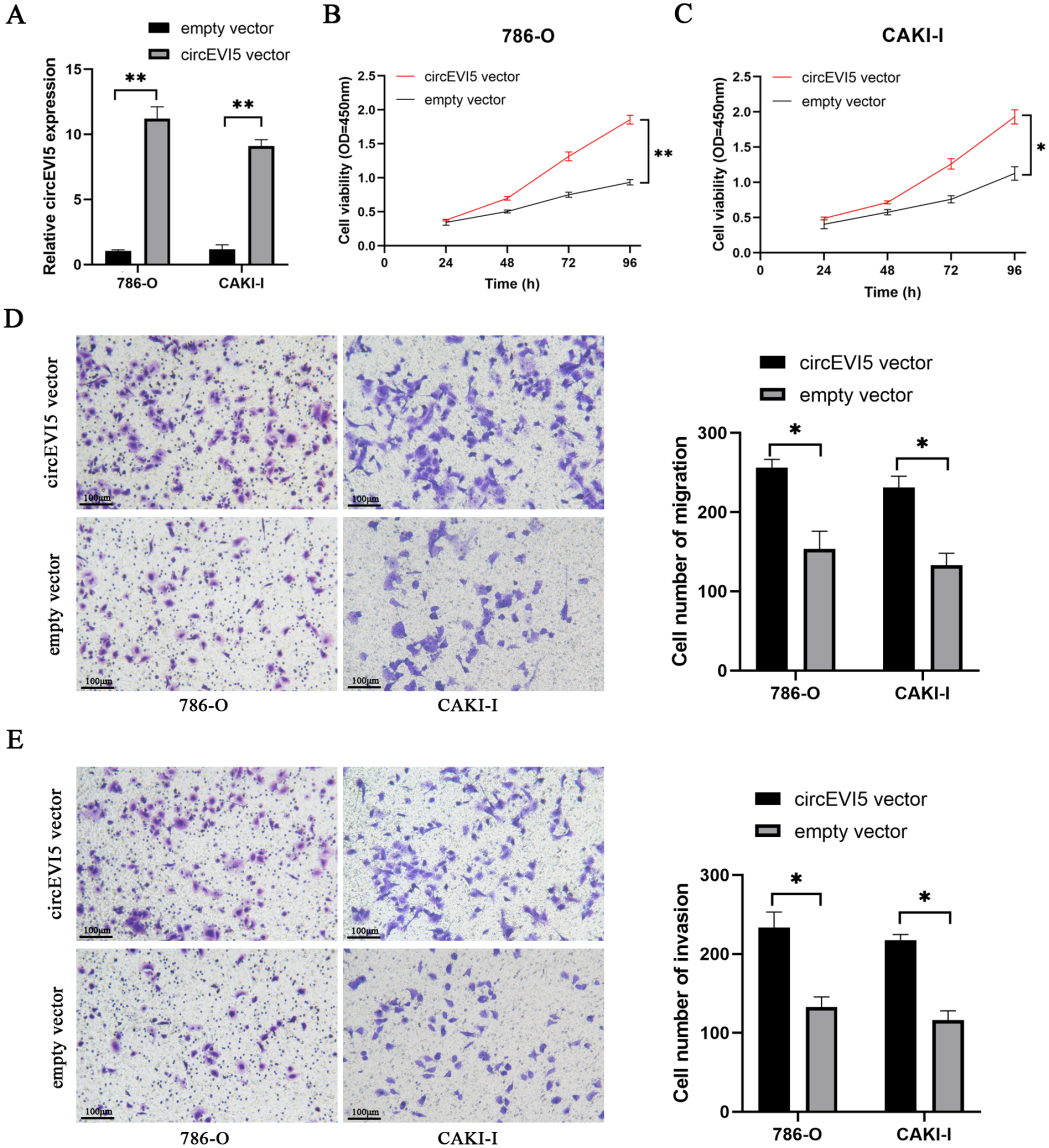
Overexpression of circEVI5 promotes proliferation, migration, and invasion of RCC cells**. (A)** qRT-PCR validation of circEVI5 overexpression efficiency in 786-O and CAKI-I cells transfected with circEVI5 overexpression plasmid (circEVI5 vector) or empty vector (control). Relative circEVI5 expression levels are shown. (**B, C).** CCK-8 assays were performed to assess cell proliferation at indicated time points (24, 48, 72, 96 h) in 786-O **(B)** and CAKI-I **(C)** cells transfected with circEVI5 vector or empty vector. Cell viability was measured by OD at 450 nm. **(D, E)** Transwell assays were conducted to evaluate migration **(D)** and invasion **(E)** abilities of 786-O and CAKI-I cells after circEVI5 overexpression or empty vector control. Representative images of crystal violet-stained cells are shown (scale bar: 100 μm). *P< 0.05, **P< 0.01 versus empty vector group.

The qRT-PCR assay analysis revealed significantly elevated expression of circEVI5 in five RCC cell lines (A498, 786-O, ACHN, CAKI-1, and OS-RC-2) compared to the normal renal epithelial cell line HK-2 (**Figure 2A**). To investigate the biological functional of circEVI5, three siRNAs targeting circEVI5 were screened. The qRT-PCR assay confirmed that all siRNAs effectively suppressed circEVI5 expression in A498 and OSRC-2 cells, with si-circEVI5–3 exhibiting the most potent silencing effect (**Figure 2B**). Consequently, si-circEVI5–3 was selected for subsequent experiments. Functional assays demonstrated that circEVI5 knockdown markedly inhibited RCC progression. CCK-8 assays showed a significant decrease in cell viability in si-circEVI5-transfected A498 and OSRC-2 cells compared to the si-NC group (**Figures 2C, D**). Transwell assays further revealed that circEVI5 silencing dramatically impaired both migration and invasion capacities of A498 and OSRC-2 cells (**Figures 2G, H**). To further investigate the functional role of circEVI5 in RCC, gain-of-function experiments were performed by overexpressing circEVI5 in 786-O and CAKI-I cells, which showed relatively low endogenous circEVI5 expression. As shown in **Figure 3A**, transfection with the circEVI5 vector resulted in a marked increase in circEVI5 expression compared to the empty vector control in both cell lines, confirming successful overexpression. CCK-8 assays revealed that circEVI5 overexpression significantly enhanced cell proliferation in both 786-O and CAKI-I cells at 48, 72, and 96 hours post-transfection (**Figures 3B, C**). Furthermore, Transwell assays demonstrated that ectopic expression of circEVI5 significantly promoted cell migration (**Figure 3D**) and invasion (**Figure 3E**) in both RCC cell lines. These results collectively indicate that circEVI5 acts as an oncogenic driver in RCC by enhancing cell proliferation, migration, and invasion.

### *In vivo* silencing of circEVI5 suppressed RCC tumor growth

To validate the oncogenic role of circEVI5 *in vivo*, a subcutaneous xenograft tumor model was established by injecting si-NC- or si-circEVI5-transfected A498 cells into nude mice. Tumor volume measurements over 5 weeks revealed a significant reduction in tumor growth in the si-circEVI5 group compared to the si-NC control (**Figure 2E**). Consistent with this, tumors resected at the endpoint (5 weeks post-injection) exhibited markedly smaller sizes and lower weights in the si-circEVI5 group (**Figure 2F**). These findings further corroborated that circEVI5 knockdown effectively attenuates RCC tumorigenesis *in vivo*.

### CircEVI5 acts as a molecular sponge for miR-433 in RCC

**Figure 4:**
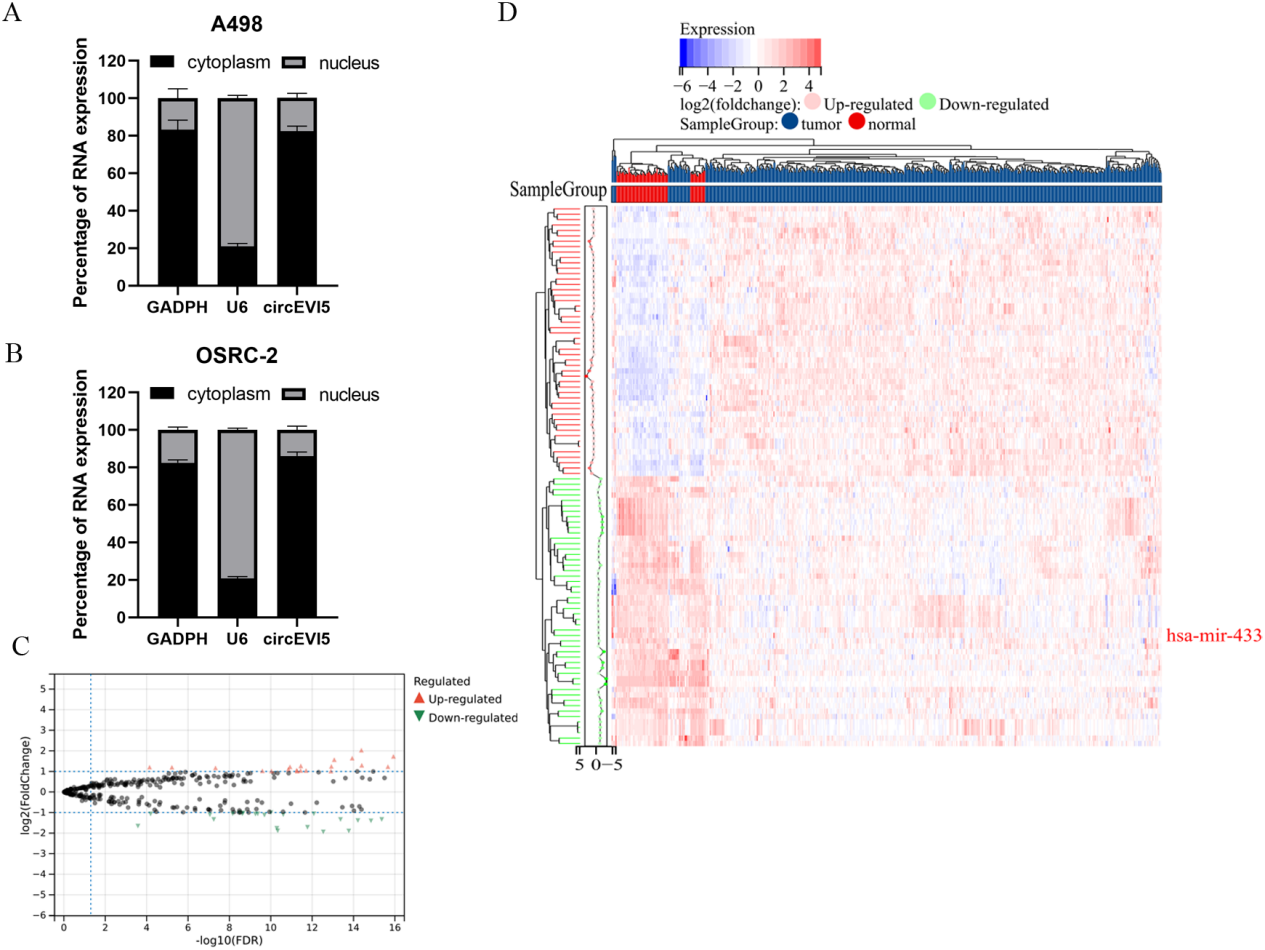
circEVI5 is enriched in the cytoplasm and associates with miRNA dysregulation in RCC. **(A, B)** the subcellular localization of circEVI5 in A498 and OSRC-2 cells were determined using cellular RNA fractionation assay. **(C)** volcano plot displayed differentially expressed miRNAs (DEmiRNAs) in RCC samples from TCGA-KIRC cohort. **(D)** the top 50 DEmiRNAs were selected for hierarchical clustering analysis. GAPDH, glyceraldehyde 3‐phosphate dehydrogenase; FDR, false discovery rate.

**Figure 5:**
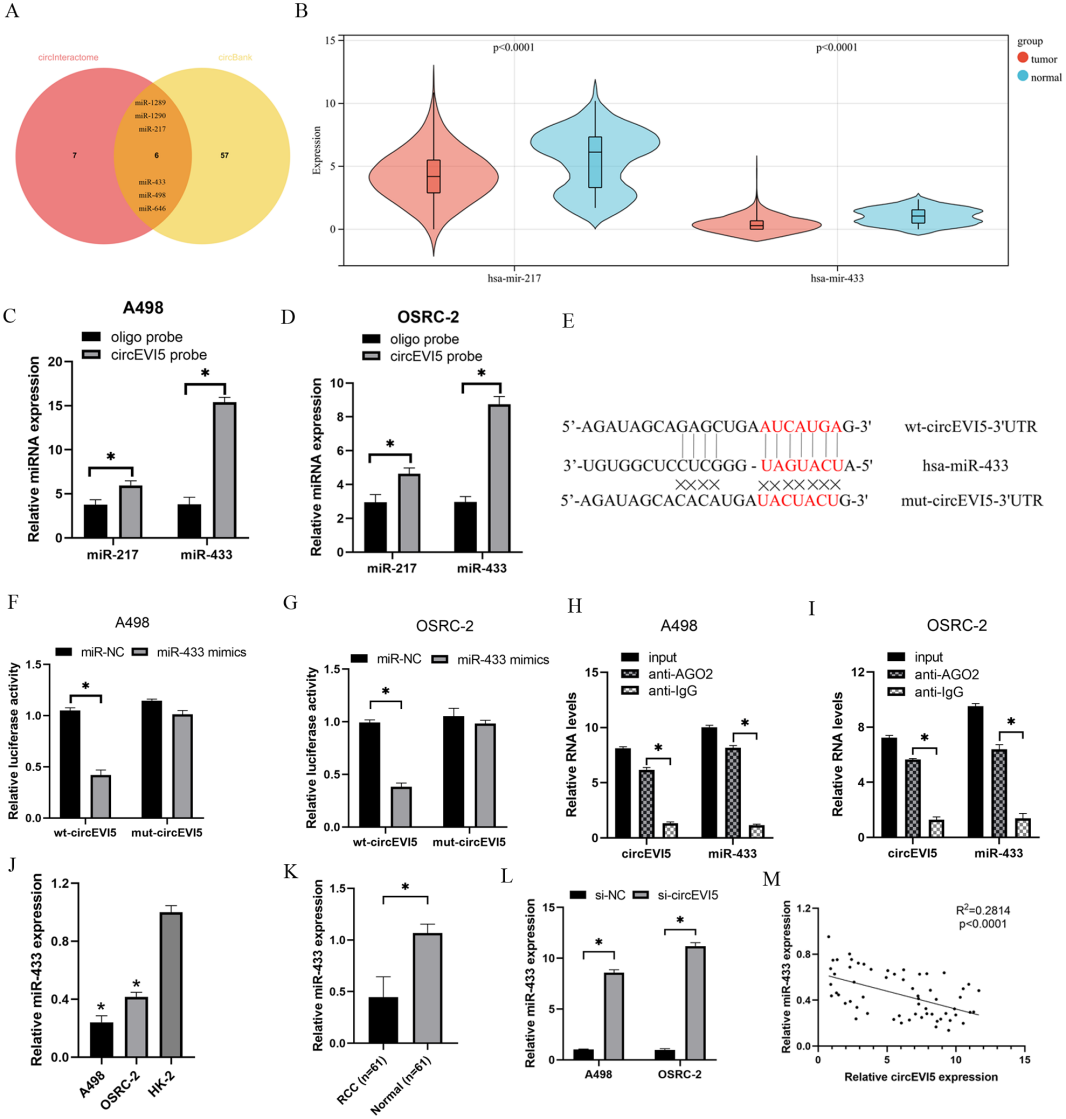
circEVI5 functions as a miR-433 sponge in RCC. **(A)** the venn diagram illustrated the circEVI5-targeted miRNAs predicted by the circInteractome and circBank databases. **(B)** the relative miR-217 and miR-433 expression was explored in TCGA-KIRC RCC tissues and adjacent non-tumor tissues. (**C, D).** miR-217 or miR-433 were pulled down in A498 and OSRC-2 cells by using a biotinylated probe specifically targets the back-splice junction of circEVI5. **(E)** diagrammatic sketch illustrated binding sites of circEVI5 and miR-433. **(F, G)** luciferase reporter assay showed miR-433 mimics significatly reduced luciferase activity of wt-circEVI5 reporter in A498 and OSRC-2 cells. **(H, I)** RIP assays demonstrated that circEVI5 and miR-433 were enriched in the miRNPs pulled down by anti-AGO2 in A498 and OSRC-2 cells. **(J, K)** miR-433 expression was inhibited in RCC cell lines and tissues. **(L)** circEVI5 inhibition upregulated miR-433 expression in A498 and OSRC-2 cells. **(M)** a significant inverse correlation was observed between circEVI5 and miR-433 expression levels in RCC tissues. *P<0.05. mut: mutant; wt: wild type.

To dissect the molecular mechanism of circEVI5, we first determined its subcellular localization. Cellular RNA fractionation assays demonstrated predominant cytoplasmic enrichment of circEVI5 in A498 and OSRC-2 cells (**Figures 4A, B**), suggesting its potential role as a miRNA sponge. To identify candidate miRNAs interacting with circEVI5, we integrated bioinformatic predictions and TCGA-KIRC data analysis. Volcano plot and hierarchical clustering analysis identified 143 differentially expressed miRNAs (DEmiRNAs) in TCGA-KIRC RCC tissues (**Figures 4C, D**). Intersection of circInteractome and circBank predictions yielded 6 candidate miRNAs (**Figure 5A**), among which only miR-217 and miR-433 were significantly downregulated in TCGA-KIRC tumor tissues (**Figure 5B**). RNA pull-down assays demonstrated that both miR-217 and miR-433 were significantly enriched by the circEVI5-specific probe designed against the back-splice junction (**Figures 5C, D**). In A498 cells, the relative enrichment of miR-433 was 15.4, which was approximately 2.6-fold higher than that of miR-217 (5.95; *P* < 0.05; **Figures 5C, D**). This prompted us to focus on miR-433. A conserved binding site for miR-433 was identified within the circEVI5 sequence (**Figure 5E**). Luciferase reporter assays confirmed that miR-433 mimics specifically suppressed the activity of wild-type circEVI5 reporter, but not the mutant construct lacking the miR-433 seed region (**Figures 5F, G**). RIP assays further validated the co-enrichment of circEVI5 and miR-433 in Ago2-containing miRNPs (**Figures 5H, I**), confirming their direct interaction within the RNA-induced silencing complex. Notably, miR-433 was consistently downregulated in RCC cell lines and clinical tissues (**Figures 5J, K**), and circEVI5 knockdown significantly restored miR-433 expression (**Figure 5L**). A strong inverse correlation was observed between circEVI5 and miR-433 levels in clinical RCC tissues (R² = 0.2814; **Figure 5M**), further supporting their functional interplay.

### GBP2 is a direct target of miR-433 and correlates with poor prognosis in RCC

**Figure 6:**
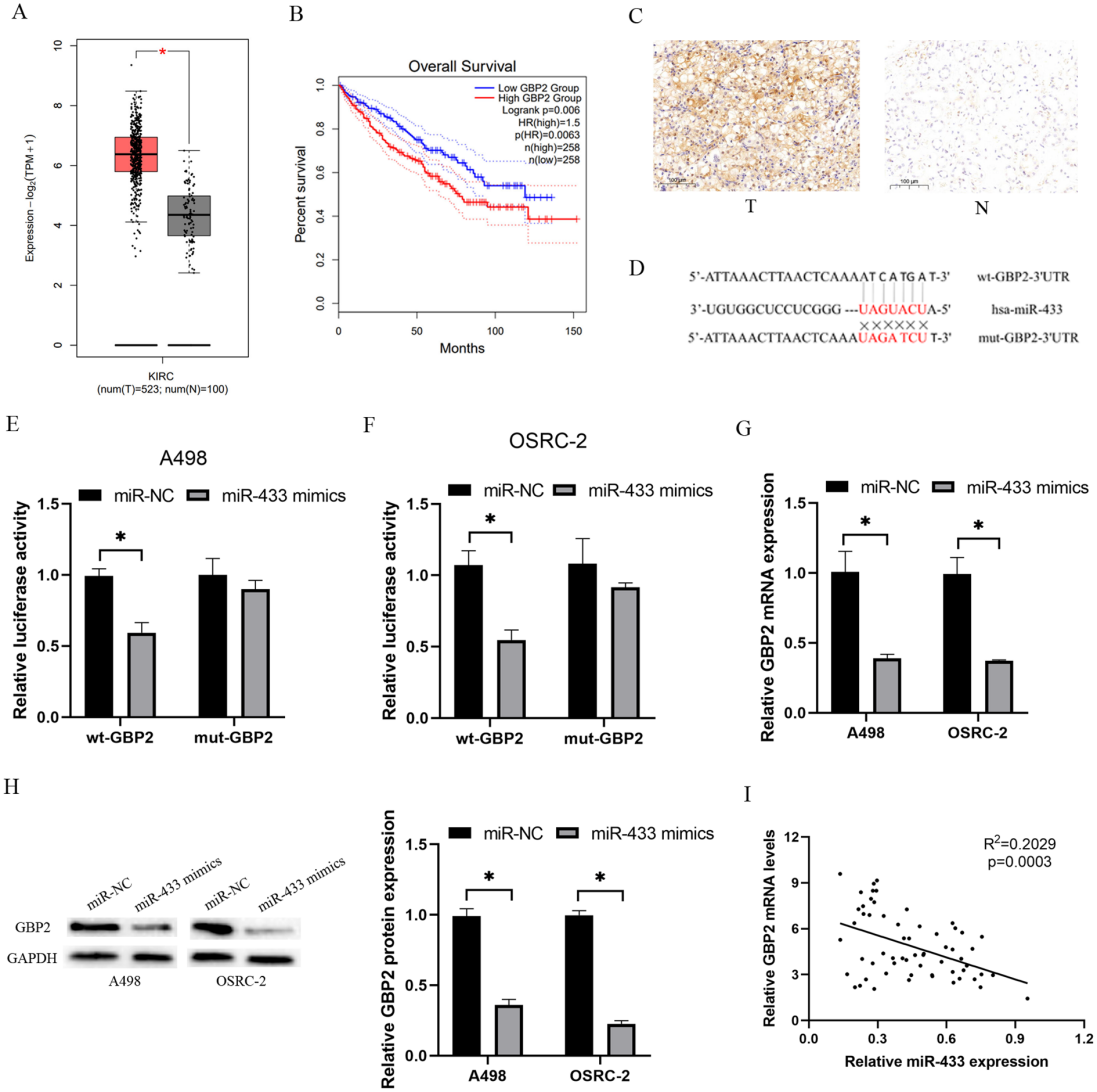
miR-433 directly targets the 3’UTR of GBP2. **(A)** GPB2 level was overexpressed in RCC tissues (n=523) compared to adjacent normal tissues (n=100) form the TCGA-KIRC samples. **(B)** high GBP2 expression correlated with reduced overall survival in TCGA-KIRC cohort. **(C)** GBP2 expression in RCC tissues and adjacent normal tissues was determined by IHC. **(D)** the binding sites of miR-433 and GBP2 3’UTR. **(E, F)** luciferase reporter assay showed miR-433 mimics significatly reduced luciferase activity of wt-GBP2 reporter in A498 and OSRC-2 cells. **(G, H)** miR-433 mimics significatly reduced GBP2 expression both in mRNA and protein levels. **(I)** a significant inverse correlation was observed between miR-433 and GBP2 mRNA expression levels in RCC tissues. *P<0.05. T, RCC tissues; N, matched non-tumor tissues; mut: mutant; wt: wild type.

Initial bioinformatics prediction using TargetScan, miRTarBase, and miRDB identified 13 putative target genes of miR-433 (MAPK8, HIVEP1, CREB1, STK38, CHD9, KRAS, BRWD1, AZIN1, ALG10B, GBP2, COX6B1, GRB2, WDR45B). Analysis of TCGA-KIRC data revealed that GBP2 expression levels were significantly elevated in RCC tissues (n = 523) compared to adjacent normal tissues (n = 100) (**Figure 6A**). Patients with high GBP2 expression exhibited markedly shorter overall survival than those with low GBP2 levels (**Figure 6B**). Although multiple miR-433 targets were predicted, GBP2 emerged as the prime oncogenic driver in RCC based on its prominent expression alteration and strong prognostic value. Consistently, immunohistochemistry (IHC) confirmed intense GBP2 protein staining in RCC tissues, whereas adjacent normal renal tissues showed weak expression (**Figure 6C**). To investigate whether GBP2 is a direct target of miR-433, we identified a conserved binding site for miR-433 within the 3’-UTR of GBP2 (**Figure 6D**). Dual-luciferase reporter assays demonstrated that miR-433 mimics significantly suppressed the activity of wild-type GBP2 3’-UTR reporter, but not the mutant construct (**Figures 6E, F**). Furthermore, miR-433 overexpression in A498 and OSRC-2 cells led to a pronounced decrease in both GBP2 mRNA and protein levels (**Figures 6G, H**), suggesting that miR-433 represses GBP2 expression at the post-transcriptional level. Clinically, a significant inverse correlation was observed between miR-433 and GBP2 mRNA levels in RCC tissues (**Figure 6I**), reinforcing the regulatory relationship between miR-433 and GBP2. These findings collectively establish GBP2 as a functional downstream effector of miR-433, contributing to RCC progression and adverse clinical outcomes.

### CircEVI5 promotes RCC progression via miR-433/GBP2 oncogenic signaling

**Figure 7:**
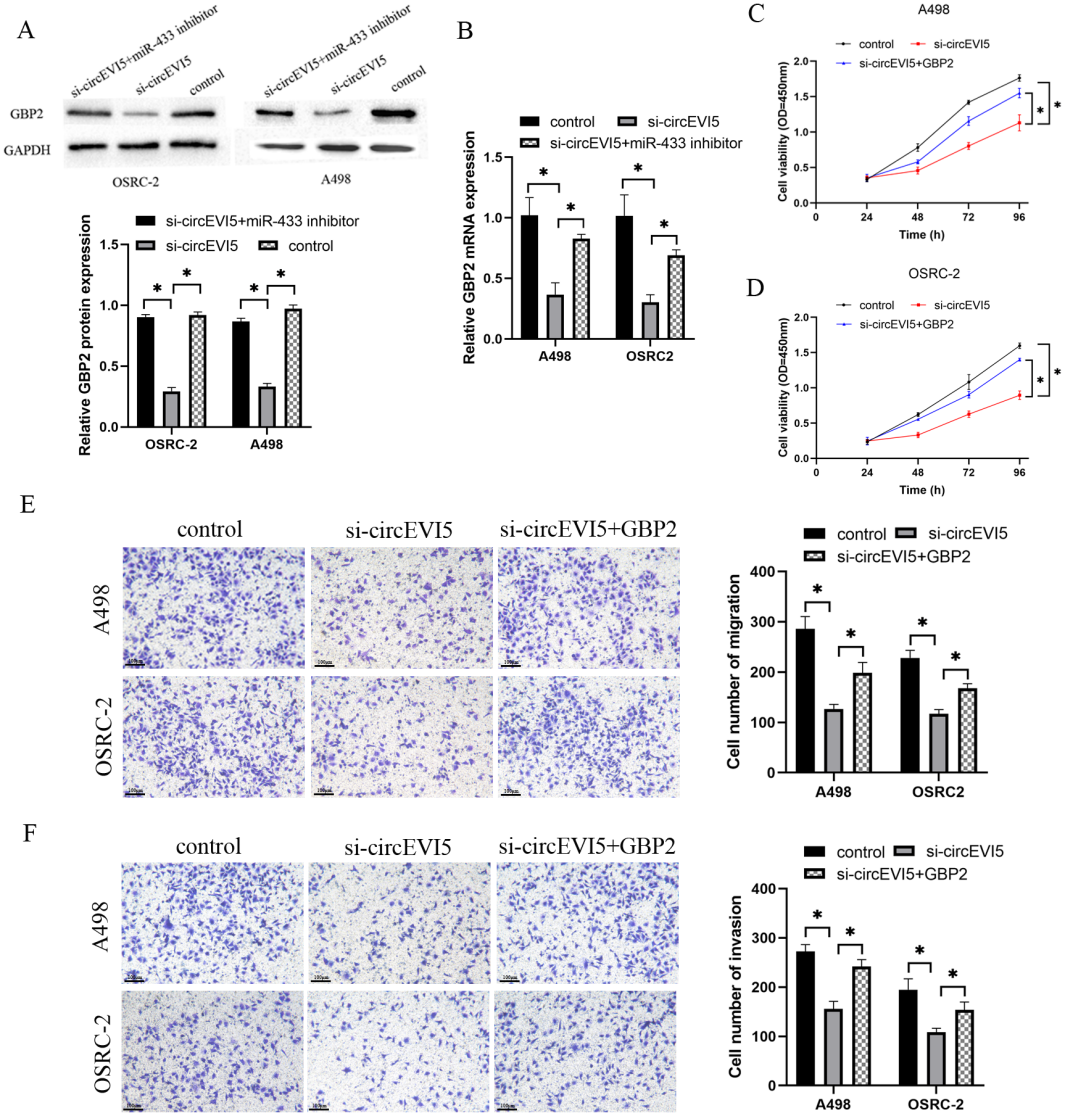
circEVI5 promotes RCC progression via miR-433/GBP2 oncogenic signaling. **(A, B)** After transfection with si-circEVI5 with or without miR‐433 inhibitor, GBP2 protein and mRNA expression were detected in A498 and OSRC-2 cells. **(C, D)** circEVI5 downregulation (si-circEVI5) inhibited A498 and OSRC-2 cells viability measured by CCK8 assay, which could be reversed by recombinant human GBP2 protein. **(E, F)** recombinant human GBP2 protein reversed si-circEVI5 inhibiting effect on A498 and OSRC-2 cells migration and invasion measured by Transwell assay. Scale bar: 100 μm.

To validate the functional linkage of the circEVI5/miR-433/GBP2 axis, rescue experiments were performed. Western blot analysis showed that circEVI5 knockdown (si-circEVI5) significantly reduced GBP2 protein expression in A498 and OSRC-2 cells, whereas co-transfection with miR-433 inhibitor effectively restored GBP2 levels (**Figure 7A**). Consistently, qRT-PCR confirmed that miR-433 inhibition reversed the si-circEVI5-induced downregulation of GBP2 mRNA (**Figure 7B**), indicating that circEVI5 regulates GBP2 through miR-433 sponge mechanism. Functional rescue assays further demonstrated that recombinant human GBP2 protein (rhGBP2) counteracted the tumor-suppressive effects of circEVI5 silencing. In CCK-8 assays, rhGBP2 (Cat. No. ab276602, Abcam) treatment (50 ng/mL for 48 h) partially restored the viability of si-circEVI5-transfected cells (**Figures 7C, D**). Similarly, Transwell assays revealed that rhGBP2 rescued the impaired migration and invasion capacities caused by circEVI5 knockdown (**Figures 7E, F**). These data collectively establish that circEVI5 exerts its oncogenic role in RCC by sequestering miR-433 to derepress GBP2 expression, thereby promoting tumor proliferation and metastasis.

## Discussion

CircRNAs were once considered to arise as a consequence of splicing errors (19). In recent years, however, there has been an explosive growth in research dedicated to circRNAs. CircRNAs have been identified as key players in normal cell differentiation, tissue homeostasis and disease progression, and their expression typically does not correlate with that of their host genes. This indicates that circRNAs are not merely stable byproducts of mRNA splicing, but rather functional transcripts derived from alternative splicing and participate in diverse regulatory pathways. Sequence conservation analysis has also confirmed that circRNAs fulfil important non-coding biological functions. Circ_001569 was shown to promote the proliferation and invasion of colorectal cancer by acting as a ‘sponge’ for miR-145 (20). CircPTPRA, as a protein sponge, was shown to bind with the key N6-methyladenosine (m^6^A) reader of insulin-like growth factor 2 mRNA-binding protein 1 (IGF2BP1) consequently blocking the recognition of IGF2BP1 by m^6^A-modified RNAs (21). In addition, circFNDC3B was shown to suppress both the progression and epithelial-mesenchymal transition of colon cancer via encoding a novel protein, circFNDC3B-218aa (22). Numerous other studies have reported on the biological functions of circRNAs in malignant tumors (11–15, 23).

Despite the numerous advances in surgical techniques and therapeutic drugs for RCC that have emerged in recent years, the overall outcomes remain unsatisfactory. Therefore, the continued exploration of novel prognostic biomarkers and therapeutic targets in RCC is of critical importance. In this study, we identified circEVI5 as a novel oncogenic driver in RCC that promotes tumor progression by functioning as a competing endogenous RNA (ceRNA) for miR-433, thereby derepressing its downstream target GBP2. After searching the PubMed, Web of Science and Google Scholar databases, we only found one study on the prognostic value of circEVI5 in gastric cancer (15). Interestingly, the study found that circEVI5 was conversely downregulated in gastric cancer tissues and cells, and that a low expression level of circEVI5 was correlated with significantly reduced relapse-free survival rates. These results indicated that circEVI5 may exhibit tissue-specific expression characteristics. CircHIPK3 was also shown to be overexpressed in epithelial ovarian cancer (24), gastric cancer (25) and prostate cancer (26). These studies confirmed that circHIPK3 could promote cell proliferation and invasion of prostate cancer through sponging miR-193a-3p and regulating the expression of myeloid leukemia 1 (MCL1). However, another study (27) showed that circHIPK3 levels were significantly downregulated in bladder cancer, and that low expression of circHIPK3 promoted autophagy in bladder cancer cells. The divergent tissue-specific expression patterns of circRNAs in tumors often suggest distinct biological functions and molecular mechanisms.

Our RNA pull-down and RIP assays confirmed the direct interaction between circEVI5 and miR-433, while luciferase reporter experiments validated GBP2 as a miR-433 target. This regulatory axis is further supported by the strong inverse correlation between miR-433 and GBP2 expression in clinical specimens. These findings echo prior studies showing that circRNAs such as circEHD2, circPCNXL2, and circSDHC promote RCC progression via miRNA sponging and downstream oncogene activation (28–30). He et al. reported that circEHD2 was upregulated in metastatic RCC tissues and serum extracellular vesicles (EVs) (28). Its silencing suppressed tumor growth both *in vitro* and *in vivo*. Mechanistically, FUS facilitated circEHD2 cyclization, enabling circEHD2 to complex with YWHAH, which bridged YAP to the SOX9 promoter, driving sustained SOX9 activation. Additionally, hnRNPA2B1 mediated the packaging of circEHD2 into EVs. The secreted EVs were then taken up by fibroblasts and induced their transformation into cancer-associated fibroblasts (CAFs). Activated CAFs promoted RCC metastasis via IL-6 and other pro-tumor cytokines. While circEVI5 and circEHD2 both exemplify the pro-tumorigenic roles of circRNAs in RCC, their distinct mechanisms—miRNA sponging versus transcriptional complex recruitment and EV-mediated stromal remodeling—highlight the complexity of circRNA biology. These differences underscore the need for context-specific therapeutic approaches while reinforcing the overarching relevance of circRNAs as biomarkers and targets in RCC management. Zhou et al. also identified a novel circRNA, circPCNXL2, which exhibited significant upregulation in ccRCC through circular RNA microarray analysis (29). Elevated circPCNXL2 levels correlated with poorer overall survival in ccRCC patients. Functional investigations demonstrated that circPCNXL2 knockdown suppressed RCC proliferation and invasion *in vitro*, while attenuating tumor growth *in vivo*. Mechanistically, circPCNXL2 acted as a miR-153 molecular sponge to modulate ZEB2 expression during RCC progression. Notably, miR-153 inhibitors reversed the anti-tumor effects of circPCNXL2 suppression on RCC cell proliferation and invasion. These findings collectively reveal that circPCNXL2 promotes RCC progression through the miR-153/ZEB2 axis, positioning it as a potential therapeutic target for ccRCC intervention. These parallel findings collectively underscore the complexity of circRNA-mediated regulation in RCC while highlighting context-dependent variations in miRNA wiring and effector pathways. The convergence on ceRNA mechanisms across independent studies strengthens the biological plausibility of this regulatory model, yet the divergence in specific molecular partners emphasizes the need for personalized therapeutic strategies targeting distinct circRNA networks in RCC subtypes. Notably, GBP2 overexpression in RCC tissues and its association with reduced survival highlight its role as a critical effector in this pathway. The rescue experiments demonstrated that miR-433 inhibition or rhGBP2 protein reversed the tumor-suppressive effects of circEVI5 knockdown, underscoring the axis’s functional relevance. These results suggest that silencing circEVI5 or restoring miR-433 activity could be viable therapeutic strategies. This aligns with recent advancements in RNA-based therapies, such as personalized cancer vaccines targeting tumor-specific neoantigens (31), and highlights the potential of combining circRNA-targeted approaches with immunotherapies like nivolumab/ipilimumab, which have shown promise in RCC (32). However, several challenges still remain, including the optimization of delivery systems for RNA therapeutics and the elimination of potential off-target effects.

While our study provides robust evidence for the circEVI5/miR-433/GBP2 axis, certain limitations should be acknowledged. First, the functional impact of circEVI5 on other miRNAs or pathways was not explored, leaving room for future multi-omics investigations. Due to the overrepresentation of ccRCC in our cohort (88.5%), the clinical implications of the circEVI5/miR-433/GBP2 axis highlighted in this study should be interpreted as principally relevant to ccRCC. Future validation in larger multi-subtype cohorts is warranted to assess its utility in rare RCC variants. Second, the *in vivo* efficacy of therapeutic interventions targeting this axis requires validation in patient-derived xenograft models. Preliminary therapeutic validation using antisense oligonucleotides or small molecules (e.g., antisense oligonucleotide [ASO]-mediated circEVI5 silencing or GBP2 inhibition *in vivo*) targeting this pathway would substantially strengthen the translational potential of our findings. Thirdly, due to the delayed implementation of the WHO/ISUP nuclear grading system in the pathology department of our hospital, grade data (G1–G4) were not available for the majority of patients in our cohort. Consequently, we were unable to include WHO/ISUP grade in the correlation analysis (**Table 2**) or the multivariate Cox regression model (**Table 3**). As nuclear grade is an established prognostic factor in RCC, its omission may potentially confound the independent prognostic value of circEVI5. Future prospective studies with complete WHO/ISUP grading data are warranted to validate our findings. Finally, the molecular mechanisms by which GBP2 drives RCC metastasis-such as its role in modulating immune evasion or extracellular matrix remodeling-warrant further study. In subsequent studies, we propose to systematically delineate GBP2-driven oncogenic signaling by integrating *in vitro* and *in vivo* approaches, including (1) multiplex cytokine profiling to evaluate GBP2-mediated immune modulation (e.g., PD-L1, IL-6, TGF-β); (2) NF-κB/STAT3 dual-luciferase reporter assays to quantify pathway activity upon GBP2 manipulation; and (3) CRISPR-based screening to map functional dependencies between GBP2 and key survival pathways (e.g., PI3K/AKT, MAPK).

## Conclusions

In conclusion, our work delineates a previously unrecognized circRNA-driven pathway in RCC and positions circEVI5 as a central player in miR-433/GBP2-mediated oncogenesis. These insights not only deepen the mechanistic understanding of RCC progression but also pave the way for translational strategies aimed at disrupting this axis to improve clinical outcomes.

## Data Availability

The datasets presented in this study can be found in online repositories. The names of the repository/repositories and accession number(s) can be found in the article/supplementary material.
